# Mid-Range Inhomogeneity of Eukaryotic Genomes

**DOI:** 10.1100/tsw.2011.82

**Published:** 2011-04-05

**Authors:** Larisa Fedorova, Alexei Fedorov

**Affiliations:** Department of Medicine, University of Toledo, Health Science Campus, Toledo, Ohio, USA

**Keywords:** pattern, DNA structure, CpG islands, isochore, triplex, Z-DNA, A-DNA, H-DNA

## Abstract

Multicellular eukaryotic genomes are replete with nonprotein coding sequences, both within genes (introns) and between them (intergenic regions). Excluding the well-recognized functional elements within these sequences (ncRNAs, transcription factor binding sites, intronic enhancers/silencers, etc.), the remaining portion is made up of so-called “dark” DNA, which still occupies the majority of the genome. This dark DNA has a profound nonrandomness in its sequence composition seen at different scales, from a few nucleotides to regions that span over hundreds of thousands of nucleotides. At the mid-range scale (from 30 up to 10,000 nt), this nonrandomness is manifested in base compositional extremes detected for each of four nucleotides (A, G, T, or C) or any of their combinations. Examples of such compositional nonrandomness are A-rich, purine-rich, or G+T-rich regions. Almost every combination of nucleotides has such enriched regions. We refer to these regions as being “inhomogeneous”. These regions are associated with unusual DNA conformations and/or particular DNA properties. In particular, mid-range inhomogeneous regions have complex arrangements relative to each other and to specific genomic sites, such as centromeres, telomeres, and promoters, pointing to their important role in genomic functioning and organization.

## INTRODUCTION

Genomic patterns on short-range scales represent various "words" composed from nucleotide "letters". Each of these words occurs many times within DNA sequences. The longest words, also known as "pyknons", comprise sequences up to 17 nucleotides (nt) long that are overabundant in the exons and introns of humans and other mammals[1,2]. The vast majority of sequences, only a little bit longer than pyknons, are unique even for the large genomes of animals and plants. For example, the complete theoretical set of 20-nt-long sequences is comprised of $4^{20}$ different words of length 20, which is just over one trillion. More than 99% of these 20-mer oligonucleotides never occur in the entire human genome (~$3*10^9$ bp). Therefore, biologists frequently use 20-mer oligonucleotides as PCR primers or hybridization probes for experimental characterization of particular genomic segments. The genomic arrangement of short sequences (<20 bp) is covered in insightful papers[3,4]. Here we consider genomic patterns longer than 30 and up to several thousands of nucleotides to be called the mid-range scale. At this mid-range, most of the sequences are unique, i.e., occur only once in the entire genome; hence, it is more appropriate to characterize or group them not by their exact sequence of nucleotides, but rather by their overall nucleotide composition, such as G+C richness, purine richness, etc. We also distinguish mid-range genomic scales from the long-range scale represented by genomic isochores, reviewed elsewhere[5]. Traditionally, G+C-rich and G+C-poor isochores are considered to be from 100 kb and longer. Recently, scientists have started to describe ultra-short isochores in the range of tens of thousands of nucleotides. In order not to interfere with isochores, we limit the length of mid-range patterns to 10,000 bases. The main focus of this paper is to show that at mid-range scales, genomes of complex eukaryotes consist of a number of different patterns and are associated with unusual DNA conformations. Some of these patterns are scarcely investigated and still wait for thorough exploration and recognition.

## G+C-RICH AND A+T-RICH REGIONS

We start considering mid-range genomic compositional patterns from the most-studied case: G+C-rich and A+T-rich regions. These G+C-rich and A+T-rich regions of various lengths from 30 to several thousand nucleotides are four to 20 times over-represented in the mammalian genomes compared to random expectation[6,7]. Among G+C-rich genomic segments, CpG islands have drawn the most public attention, due to their functional properties and involvement in gene expression regulation[8]. CpG islands are found in nearly 60% of human genes, including almost all of the housekeeping genes[8]. According to two different definitions of these islands, their length must be at least 200 or 500 bp, G+C content more than 50 or 55%, and the number of CpG dinucleotides in the islands should exceed more than twice their occurrence in other genomic regions[9,10]. CpG dinucleotides are important sites for cytosine methylation in all vertebrates and some invertebrates and plants. However, inside CpG islands, CpG dinucleotides are predominantly nonmethylated[11]. It has been shown recently that CpG dinucleotides without methylation exhibit structural abnormalities in the DNA helix. Particularly, they are one of the most frequent sites for DNA backbone cleavage by hydroxyl radicals[12,13] and during the sonication of double-stranded DNA[14]. The crucial involvement of cytosine methylation in the regulation of gene expression is well described in a number of reviews, including some recent ones[11,15,16]. Thus, we concentrate here on the other physicochemical properties of G+C-rich and A+T-rich regions.

It is well known that the A form of the DNA helix exists in high salt concentrations and in ethanol-containing solutions. However, G+C-rich regions may be present in A-form DNA even in aqueous solutions[17,18,19]. A special form of DNA that is an intermediate between A and B forms has been characterized in G+C-rich sequences with methylated cytosines[20]. In addition, short $(CpG)_n$ repeats could adopt Z-DNA (reviewed by Ho[21]). This Z-DNA is proposed to serve as a transcriptional coactivator[22].

A+T-rich regions, on the other hand, are also associated with special DNA conformations. Some of these sequences with specific distributions of A and T bases form an unusual structure known as the DNA unwinding element[23]. These elements are often associated with the origins of replication in eukaryotes and prokaryotes[24]. There are several A+T-rich simple repeats widespread in eukaryotes. Among them, $(AT)_n$ is one of the most common in animals. X-ray and NMR studies of the DNA oligomer d(ATATAT) have shown that, in addition to B-DNA, it could form an antiparallel, double-helical duplex in which the base pairing is of the Hoogsteen type[25]. The adenines in this duplex are flipped over, making the minor groove narrow and hydrophobic. This structure is very similar to the standard B-form helix with about 10 bp per turn. Theoretical analysis has demonstrated that energies of the Hoogsteen form and B form of DNA are practically identical[26]. Most recently, Chakraborty et al.[27] demonstrated that poly-dA oligonucleotides $(dA_{15})$ under acidic pH conditions could allow the formation of a double-helical parallel-stranded duplex held together by reversed Hoogsteen-type $AH^+$-$H^+A$ base pairs.

A+T-rich regions presumably have several important cellular functions. First, the most indicative compositional characteristic of scaffold/matrix-attached regions is that they are A+T rich [28]. Second, centromere DNA of diverse animals, plants, and fungi always contain A+T-rich regions[25,29].

## PURINE/PYRIMIDINE-RICH REGIONS AND H-DNA TRIPLEX

All combinations of nucleotide pairs, except G+C and A+T, have strand asymmetry. For example, if one strand is enriched by purines (R), the complementary strand is enriched by pyrimidines (Y). Therefore, R-rich and Y-rich sequences and T+G-rich and A+C-rich sequences are physically the same loci, yet represent complementary strands. From here on, we will consider them together and refer to them as R/Y-rich and T+G/A+C-rich, respectively.

Since 1957, it has been shown that complementary DNA strands, one of which is R-rich and another Y-rich, can form three-stranded helical structures or triplexes[30]. Intramolecular triplexes, known also as H-DNA, materialize under certain conditions, like supercoiling, when half of the DNA duplex may dissociate into single strands and one of the stand-alone strands can interact via Hoogsteen base pairing with the remaining Watson-Crick DNA duplex along its major groove, forming a triplex structure. The remaining stand-alone strand stays unpaired. An example of a DNA triplex is shown in Fig. 1. There are four kinds of H-DNA depending on strand type and orientation[31]. One type of H-DNA forms under acidic conditions when the stand-alone Y-rich strand interacts with the R-rich strand of the remaining duplex. Particularly, thymines of the stand-alone strand interact with adenosines of the A-T Watson-Crick pairs of the duplex via Hoogsteen hydrogen bonding, while cytosines of the stand-alone strand interact with guanines of G-C Watson-Crick pairs. Due to this base match requirement for the assembly of this kind of triplex, the sequences of the Y-rich stand-alone strand and the Y-rich strand in the duplex should have sequence mirror symmetry. (Here is an example of two sequences with mirror symmetry: 5-TAGTTCC-3 and 5-CCTTGAT-3.) In many R/Y-rich regions of the genomes, such mirror symmetry has been observed. For example, a 2.5-kb R-rich sequence of the 21$^{st}$ intron of the human PKD1 gene has 23 mirror repeats that form H-DNA[32,33]. Another kind of intramolecular triplex can be formed at neutral pH and requires bivalent cations for stability. It is formed by the interaction of the R-rich stand-alone strand with the remaining duplex via Hoogsteen bonding. It does not require strong mirror symmetry within its sequences, since the adenines of the stand-alone R-rich strand could interact with the A-T pair of the duplex or with the G-C pair[34].

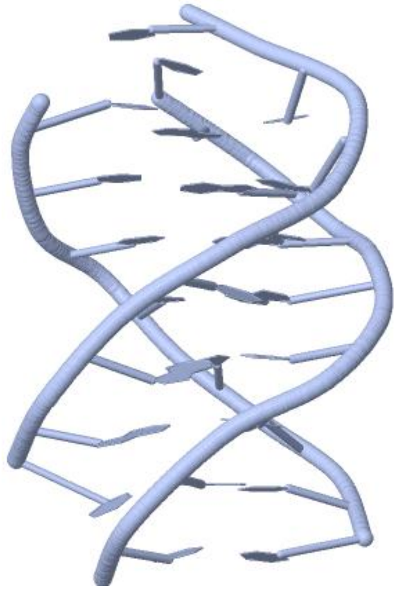

**FIGURE 1.** Cartoon of 3D structure of a purine-purine-pyrimidine DNA triplex containing G-GC and T-AT triples. This picture is a snapshot of the structure with the identifier 134D obtained from the Protein Data Bank. The structure was resolved using a combined NMR and molecular dynamics approach by Radhakrishnan and Patel[35].

There are several documented functions of H-DNA. It is well established that H-DNA could exist *in vivo* under certain conditions. Various experimental methods for the characterization of H-DNA have been reviewed by Jain et al.[31] and Wang et al.[36]. Single-stranded DNA not participating in the triplex is accessible to S1-nuclease cleavage. Eukaryotic genomes contain many S1-nuclease–sensitive sites within runs of homopurine sequences. These segments of single-stranded DNA are frequently involved in the recombination of homologous DNA and, thus, are sites for genetic instability. Different schemes of recombination involving H-DNA have been described by Jain et al.[31]. Bacolla et al.[37] characterized nearly 3,000 homopurine tracks in the human genome longer than 100 nt. They supported evidence for these tracks in promoting recombination and association with higher rates of mutations. In addition, stable H-DNA structures are able to block transcription and replication. Jain and coauthors surveyed the evidence for how H-DNA influences the activity of DNA and RNA polymerases. Finally, Goni and others[38] performed a large-scale bioinformatic analysis of the distribution of short R-rich sequences in the human genome. They demonstrated that short R-rich sequences are several times more abundant in the downstream promoter regions compared to other regions and to random expectation models. These short R-rich sequences hold evolutionary conservation between human and mouse, yet they likely are not direct targets for transcription factors. Goni and coauthors have suggested that these sequences act as pacing fragments in promoter regions and help in the correct positioning of transcription factors.

## G+T-RICH/A+C-RICH REGIONS

Recall that the complementary strands of G+T-rich regions are naturally A+C-rich regions. They coexist with each other and we consider them interchangeable with respect to their description in the literature. According to nucleic acid nomenclature, G or T nucleotides are also known as *Keto* or K, while A or C are known as *Imino* or M. Thus, sometimes these regions are referred to as K.M tracks or motifs[39]. Bechtel and coauthors demonstrated that G+T regions are about five times more abundant in the mammalian genomes compared to random expectation[7]. Moreover, these regions practically do not intersect with interspersed DNA repeats at all. In 2004, Yagil[39] demonstrated that K.M motifs are significantly over-represented in the genomes of diverse animals, plants, and fungi. Specifically, K.M motifs are predominant in the *Drosophila melanogaster* genome, where they outnumber other motifs such as R/Y-rich motifs. Despite their abundance, G+T-rich motifs are much less investigated than other regions with extremes in base compositions. Possible functions that could be associated to G+T-rich regions are the following. First, $(CA)_N$ simple repeats are one of the most profuse tandem repeats in mammalian genomes[40]. They also should be considered as an alternating R/Y sequence and, due to this property, associated with a Z-DNA conformation[41], which is considered in the next section. Second, C-rich regions, which could be a component of CA-rich regions, are capable of forming four-stranded intercalated molecules[42]. Third, short G+T-rich regions could represent transcription factor binding sites, such as for factor Sp1[43]. Fourth, telomeres of various eukaryotic species are represented by G+T-rich regions that form G-quadruplexes, also known as G-quartets or G-4. Quadruplexes are arranged in four-stranded structures with strands connected to each other via Hoogsteen hydrogen bonding between guanines. The G-quadruplex has been well characterized in human telomeric and related sequences with the core repetitive element TTAGGGG, and within promoters and 5´-untranslated regions of human genes whose sequences have a loose consensus of $G_{3\text{-}5}N_{L1}G_{3\text{-}5}N_{L2}G_{3\text{-}5}N_{L3}G_{3\text{-}5}$, where $N_{L1}$, $N_{L2}$, and $N_{L3}$ are loops with the length from 1 to 7 nt and variable nucleotide composition[44]. Intriguingly, G+T-rich oligonucleotides possess antiviral activities. For example, the $T_2(G_4T_2)_3$ sequence is virucidal against the herpes simplex virus[45]. At the RNA level, C+A-rich sequences within intronic segments could regulate alternative splicing by being binding sites for the hnRNP L protein[46]. The presence of C+A-rich sequences at the 3´-UTR of mRNA could regulate gene expression at the level of translation[47]. The distribution of C+A-rich sequences enriched by $(CA)_N$ imperfect repeats is highly skewed towards telomeres and minisatellites can usually be found in the vicinity as well[48]. Despite the listed properties associated with G+T-rich regions, they seem significantly underinvestigated and may yet reveal unknown important functional properties in the near future.

## ALTERNATED PURINE/PYRIMIDINE REGIONS AND Z-DNA

Left-handed antiparallel Z-DNA double-helix conformation has been first characterized in 1979 by Wang and coauthors for $(GC)_3$ repeats[49]. Detailed Z-DNA structure has been considered elsewhere[21,50]. This particular conformation is characterized by rotation of R bases that adopt *syn* form and stack over the deoxyribose ring, while Y bases do not adopt unfavorable *syn* form[21]. Thus, Z-DNA, which is characterized by an alternating pattern of *anti*-*syn* conformations, is formed by alternated R/Y sequences[51].

In 1986, Ho and others[52] developed a ZHUNT program for detection of genomic sequences with high propensity to form Z-DNA. They found a high concentration of these sequences near the transcription start sites[50,53]. Most recently, human genomic Z-DNA segments have been detected experimentally using a Z-DNA binding protein domain as a probe[54]. The authors found an abundance of Z-DNA hot spots located in centromeres of 13 human chromosomes. Z-DNA–forming sequences induce high levels of genetic instability in both mammalian and bacterial cells. These sequences could be causative factors for gene translocations found in leukemias and lymphomas[55]. The discovery of certain classes of proteins bound to Z-DNA with high affinity and specificity indicated a biological role of this structure. Yet, it is a common view that Z-DNA is an unstable conformation that is formed and disappears during particular physiological activities, such as transcription[50].

The latest version of the *Genomic MRI* package (described in detail in the next section) has a new feature that allows the detection of excesses and shortages of alternating bases, including R/Y patterns. It reveals that in mammalian genomes, there is more than 40 times the overabundance of alternating R/Y stretching over 50- to 100-bp genomic segments, where RY plus YR comprise more than 80% of all dinucleotides. A considerable portion of these alternating R/Y patterns are represented by short $(GC)_n$, $(AC)_n$, $(AT)_n$, and $(TG)_n$ repeats that can alternate with each other and be accompanied by alternating R/Y bases without strong periodic sequence pattern. For example, here is a sequence of a 50-bp segment from the third intron of a human heparanase-2 gene highly enriched with alternated R and Y bases: 5′AAATGGATGTGTGTATATATATGAAGTCGATACACACACATATACACATA3′. We showed that such alternating R/Y sequences are plentiful throughout the mammalian genomes, either inside introns or within intergenic regions.

### *Genomic MRI* Program Package

In thousands of genomic regions, the composition of A, T, C, or G content or different combinations of these bases exist at extremes far different from the average base composition. We call such compositional extremes genomic mid-range inhomogeneity (or MRI) if they stretch at least 30 bp, but <10,000 bp. To characterize genomic MRI patterns, a public computational resource (*Genomic MRI*) has been created that allows us to detect sequence regions with any type of extreme composition[7]. Using this resource, it was demonstrated that various MRI regions occupy up to a quarter of the human genome and their existence is maintained via strong fixation bias[56]. For examining mid-range sequence patterns, *Genomic MRI* programs do not characterize particular "words", but only the overall compositional content of particular base(s) that we refer to as X (X could be a single nucleotide A, G, C, or T, or any of their combinations like A+C or G+T+C, etc.). *Genomic MRI* allows us to study the distribution of X-rich regions in any sequence of interest. These X-rich MRI regions are highly over-represented in mammalian genomes for all kinds of X contexts. For instance, in the human genome, G+C-rich sequences with lengths from 100 to 200 nt are 20 times over-represented; A+T-rich sequences in the same length range are about 12 times over-represented; A+G-rich and T+C-rich sequences 10 times; and G+T-rich and A+C-rich sequences up to six times over-represented[7]. In order to measure the abundance of X-rich regions in the sequences under analysis, *Genomic MRI* compares their presence inside a specifically generated random sequence that has the same oligonucleotide distribution as the real one. This evaluation is achieved by the following computational steps. First, the short-range inhomogeneity (SRI) of a given sequence is analyzed by the SRI-*analyzer* program from the *Genomic MRI* package to create an oligonucleotide frequency table for each possible 1- to 9-nt-long "word". Then, a second program, SRI-*generator*, creates a random sequence with SRI approximating the oligonucleotide frequency table of the natural sequence. This random sequence is used further for comparison with the natural one. Finally, the third program, MRI-*analyzer*, scans a sequence under analysis and the random sequence with a window of a specified size, and checks whether the nucleotide composition of the sequence in the current window is X rich or X poor for a particular chosen combination of nucleotides (X), e.g., A, T, C, G, G+C, A+G, G+T, etc. A window is rich for the X content if its X composition is above a user-specified threshold-$X_1$, while a window is X poor if it is below another user-specified threshold-$X_2$. (Note that X-poor regions can be referred to as non–X-rich regions, e.g., G+C poor are A+T rich.) An example of MRI-*analyzer* graphical output is shown in Fig. 2, which illustrates the MRI patterns for an extra-large human intron of the dystrophin gene from chromosome X.

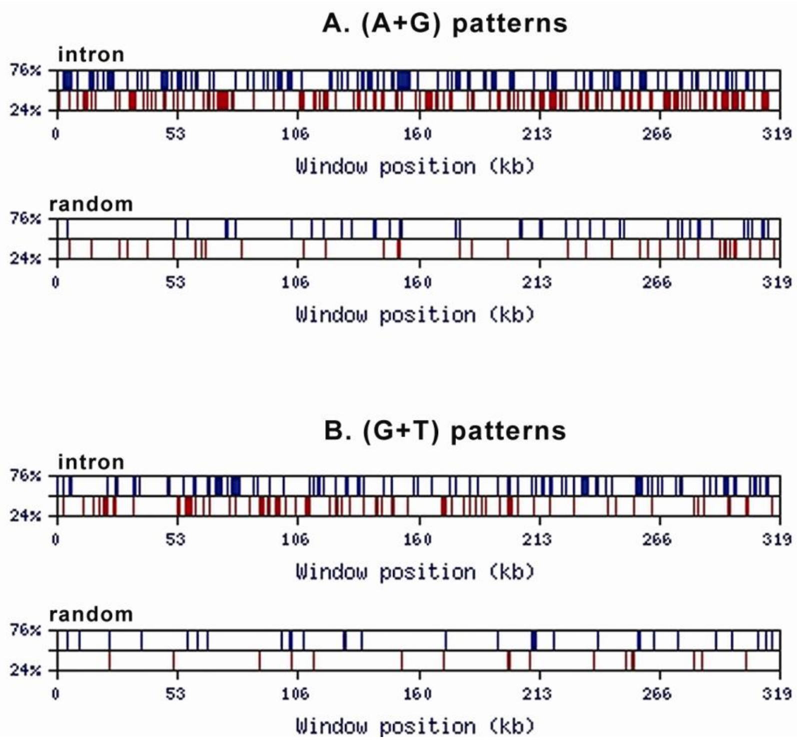

**FIGURE 2.** The graphical output of the MRI-*analyzer* program for the first intron of the dystrophin gene (marked as "intron") and also for the SRI-*generator* random sequence based on the tetramer oligonucleotide frequency table of the intron (marked as "random"). The entire sequence of the 319-kb intron and the random sequence is displayed on the x axis. Blue bars represent content-rich MRI regions on the sequence. Red bars represent content-poor MRI regions. The y axis contains upper and lower thresholds for the given content type. (A) *Genomic MRI* analysis of A+G-rich and A+G-poor (or T+C-rich) regions; (B) *Genomic MRI* analysis of G+T-rich and G+T-poor (or C+A-rich) regions.

Two scales of MRI regions should be considered: (1) regions (from 30 to 1000 bp) whose properties have been investigated in detail and for which several periodicities have been reported[57,58,59]; (2) larger regions (from 1 to 10 kb), which are one of the least-studied areas in genomic composition and where as-yet-unknown biological properties may be found. Such subdivisions are important for the proper choice of parameters for the MRI thresholds. For instance, for a 100-nt-long window, there is a vast number of regions in mammals where G+C composition is 85% or higher. However, for studying regions with a window size of around 5 kb, the upper threshold for G+C content should not be more than 65% to find the areas satisfying the criterion.

## A COMPLEX MOSAIC OF MRI PATTERNS

Different MRI regions are not randomly arranged relative to each other[6]. For example, Fig. 3 illustrates that G+C-rich regions tend to be associated in clusters. On the other hand, the distribution of A+T-rich regions is much more close to a random distribution with the exception that A+T-rich regions avoid very close proximity to each other[6]. So far, investigators have examined only individual genomic patterns. The mutual arrangement of various genomic mid-range patterns has never been thoroughly investigated. Our preliminary results suggest that within mammalian genomes, there is a complex mosaic picture of MRI regions. Modeling sequences only with one particular type of MRI compositional bias using the MRI-*generator* program from the *Genomic MRI* package has proven to not be a trivial computational task[7]. This has given us an appreciation that the reconstruction of the entire set of MRI patterns in modeling DNA sequences is an extremely challenging mission due to a complex multilayer nonrandomness in genomic sequences. In addition, genomic sequences have an intricate organization of nested patterns with respect to the clustering of particular patterns. Some features of this complex organization were described as genomic fractals in several publications[60,61,62]. This arrangement has been studied by methods such as “detrended fluctuation analysis” and a “Brownian walk” in order to uncover relationships such as power law correlations and exponential decays, which assess the scaling behavior of a system. This scaling behavior is related to fractal geometry and deals with “self-similarity”, defined as the property of resembling a subset of oneself. Earlier investigations of this kind generally confined themselves to clusters of purines and pyrimidines, but later studies have shifted to examining G+C and A+T clusters for the thermodynamic implications of their pair binding[60,61,63,64,65,66,67].

Recently, by studying the distribution of more than 4 million SNPs in the human genome and by taking into account their frequencies in the population, the influence of mutations on different MRI regions has been examined[56]. The authors demonstrated that MRI regions have comparable levels of *de novo* mutations to the control genomic sequences with average base composition. *De novo* substitutions rapidly erode MRI regions, bringing their nucleotide composition toward genome-average levels. However, those substitutions that favor the maintenance of MRI properties have a higher chance to spread through the entire population. The observed strong fixation bias for mutations helps to preserve MRI regions during evolution, indicating their potential significance to genomic operations.

On the other hand, a large portion of MRI regions could have a mechanistic origin due to the bias in frequencies of different types of mutations as well as fixation bias of these mutations. Indeed, the rate of transition mutations is generated at higher frequency than transversions, even though there are twice as many possible transversions. Moreover, the rate of particular types of mutation (e.g., A->C) is influenced by the surrounding nucleotides (context). For example, CpG dinucleotides are a hot spot for C->T and G->A changes due to methylation of the cytosines within this context. The charts for all possible human substitution frequencies within the context of a single 5´ nucleotide are presented by Zhang and Gerstein[68]. Among transversions, the highest frequency was observed for tA->tC substitutions, which is about three to four times higher than those for cT->cG, cC->cG, and tT->tA substitutions having the lowest frequencies[68]. Many sequence patterns may arise in accordance to the widely accepted neutral theory of molecular evolution without involvement of negative (purifying) and/or positive selection[69]. The

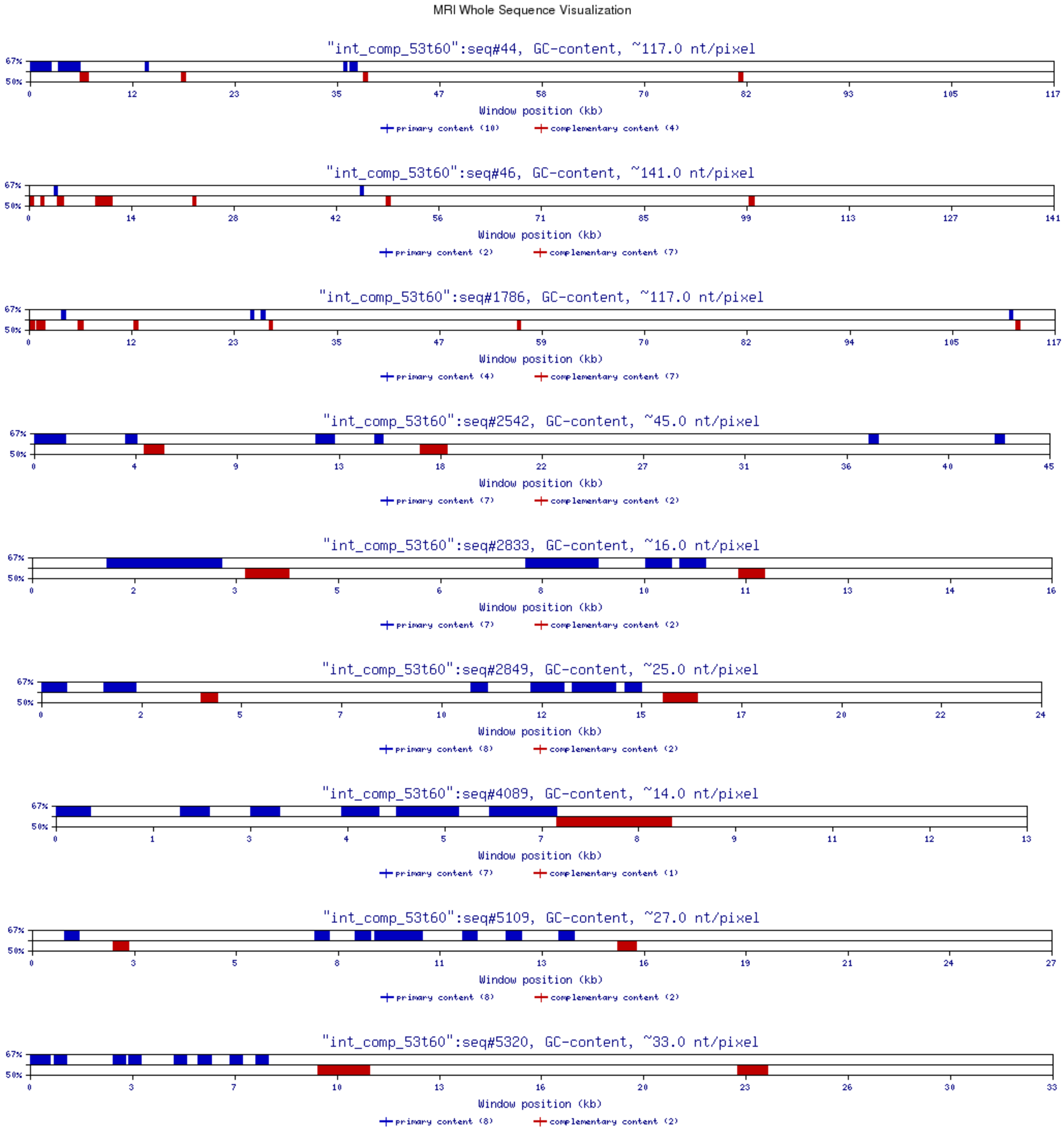

**FIGURE 3.** Visualization of G+C-rich (blue) and A+T-rich (red) MRI features in human introns using a 400-nt base window size. The scale for each sequence is independent and is given in its subheading in nucleotides per pixel. The figure represents a fragment of Fig. 17 in Bechtel[6].

mechanistic origin of genomic compositional inhomogeneity is the basis for the Biased Gene Conversion (BGC) theory for the origin of GC-rich isochores, lately detailed by Duret and Galtier[70]. Particularly, BGC theory explains GC-rich isochores due to fixation bias in favor of AT->GC mutations that occur without positive Darwinian selection. Currently, a popular view is that both selective and neutral processes drive GC content evolution in the human genome[70,71].

It is important to note that various simple repeats make up one of the widespread components of MRI regions, e.g., $(AT)_n$ repeats for A+T-rich MRI regions and $(AGG)_n$ for purine-rich regions. During evolution, simple repeats are subjected to growth via replication slippage and interchromosomal exchange[72], and hence their existence may lack functional importance. At the same time, there are up to 10 different non–B-form DNA conformations connected with simple repeats, as are listed and well illustrated by Wells[73]. Interestingly, more than 70 human genetic disorders have been associated with changes in simple repeats[73,74]. In rodents, 2.4% of their euchromatin is represented by simple repeats, which is two times bigger than the length of all their protein-coding sequences[75]. An important public toolkit is available online for the characterization of simple repeats as well as the analysis of DNA sequence complexity. Programs include Compexity, LZcomposer, OligoRep, and more[76,77].

## THE ROLE OF MRI REGIONS

Often, in the popular literature, genomes are presented as a set of texts or instructions. Such a representation implies that there should be an intelligent creature somewhere inside a cell interpreting these DNA texts. Thus, it is more appropriate to compare genomes with self-realization programs that autonomously fulfill their tasks and are able to respond to environment signals and conditions. Such programs must be extremely complicated for complex organisms, like humans, which are built from trillions of cells of hundreds of different kinds, yet sharing the same genomic sequence. There must be fundamental principles for construction and functioning of genomic programs. One of the most important is the Principle of Recursive Genome Function (PRGF) illuminated by Pellionisz[62]. The author considers the genome as an unsupervised operating system. The well-known examples of such a system are neural networks for which mathematical models describing their behavior have been developed. According to Pellionisz, “the recursive genome function is a process when at every step of development already-built proteins iteratively access sets of primary and ensuing auxiliary information packets of DNA to build constantly developing hierarchies of protein structures.” In other words, there is a crucial flow of information from proteins back to the genomic DNA. According to Pellionisz, this principle converts a genome from a *closed* to an *open* physical system and resolves the paradox of genomic entropy posed by Sanford[78]. This perspective elucidates the importance of MRI regions as specific sites for changing genomic information by proteins. Indeed, MRI regions are intricately associated with unusual DNA conformations, which in turn are binding sites for a number of proteins. These proteins could stabilize and/or initiate DNA conformation transformation and propagate the signal along neighboring DNA segments. For instance, Z-DNA binding proteins could initiate this transformation from right-handed B-DNA to the left-handed Z form. This structural transition changes the DNA supercoiling for the regional DNA landscape and additionally creates specific B-Z boundaries with flipped-over bases. Such transformation could modify, open, and/or hide some information on the genomic DNA, not only at the protein binding site, but within neighboring regions.

## CONCLUSIONS

Overall, within vast areas of previously thought “junk DNA”, represented by introns and intergenic sequences, there exists an intricate mosaic of various MRI regions with extreme base compositions. Various genomic MRI regions are tightly associated with unusual DNA conformations and must be of crucial importance for proper functioning of multicellular eukaryotes.

## ACKNOWLEDGMENTS

This work was supported by the National Science Foundation (Career MCB-0643542).

A modified version of this paper is to be published as a chapter for a book: “Advances in Genome Sequence Analysis and Pattern Discovery” 2011.
